# RELIC: a novel dye-bias correction method for Illumina Methylation BeadChip

**DOI:** 10.1186/s12864-016-3426-3

**Published:** 2017-01-03

**Authors:** Zongli Xu, Sabine A. S. Langie, Patrick De Boever, Jack A. Taylor, Liang Niu

**Affiliations:** 1 Epidemiology Branch, National Institute of Environmental Health Sciences, NIH, Research Triangle Park, NC USA; 2 Environmental Risk and Health unit, Flemish Institute for Technological Research (VITO), Mol, Belgium; 3 Faculty of Sciences, Hasselt University, Diepenbeek, Belgium; 4 Epigenetic and Stem Cell Biology Laboratory, National Institute of Environmental Health Sciences, NIH, Research Triangle Park, NC USA; 5 Division of Biostatistics and Bioinformatics, Department of Environmental Health, College of Medicine, University of Cincinnati, Cincinnati, OH USA

**Keywords:** RELIC, DNA methylation, Dye-bias correction, Illumina HumanMethylation450, Illumina MethylationEPIC, Data preprocessing

## Abstract

**Background:**

The Illumina Infinium HumanMethylation450 BeadChip and its successor, Infinium MethylationEPIC BeadChip, have been extensively utilized in epigenome-wide association studies. Both arrays use two fluorescent dyes (Cy3-green/Cy5-red) to measure methylation level at CpG sites. However, performance difference between dyes can result in biased estimates of methylation levels.

**Results:**

Here we describe a novel method, called REgression on Logarithm of Internal Control probes (RELIC) to correct for dye bias on whole array by utilizing the intensity values of paired internal control probes that monitor the two color channels. We evaluate the method in several datasets against other widely used dye-bias correction methods. Results on data quality improvement showed that RELIC correction statistically significantly outperforms alternative dye-bias correction methods. We incorporated the method into the R package ENmix, which is freely available from the Bioconductor website (https://www.bioconductor.org/packages/release/bioc/html/ENmix.html).

**Conclusions:**

RELIC is an efficient and robust method to correct for dye-bias in Illumina Methylation BeadChip data. It outperforms other alternative methods and conveniently implemented in R package ENmix to facilitate DNA methylation studies.

**Electronic supplementary material:**

The online version of this article (doi:10.1186/s12864-016-3426-3) contains supplementary material, which is available to authorized users.

## Background

DNA methylation arrays are commonly employed in large-scale epigenome-wide studies. The Illumina Infinium HumanMethylation450 BeadChip provides methylation measurements at more than 485,000 individual CpG sites [2], and its successor MethylationEPIC BeadChip provides almost twice as many sites (>850,000). Both arrays are two-color channel (Cy3-green/Cy5-red) microarrays and employ two chemical assays (Infinium I and Infinium II). Whereas Infinium I uses two probes, both labeled with the same dye (either Cy3 or Cy5), to evaluate the methylated and unmethylated states of each target CpG site, Infinium II uses a single probe per locus with the competitive binding of methylated (Cy3-green) and unmethylated (Cy5-red) alleles evaluated in different color channels. For each CpG site, the measurements of the two states (methylated and unmethylated) are intensity values (*M* and *U*) and the measurement of the methylation level, called beta value, is the ratio between methylated and unmethylated intensity and is calculated as *M*/(*M* + *U* + *c*), where *c* is an offset usually assigned a value of 100 to regularize the beta value when both *M* and *U* are small.

It was known that the two channels usually perform differently [3] with often overall higher intensity values on the red channel than the green channel. This dye-bias in intensity values can have a direct impact on the calculation of methylation beta values. Thus, a dye-bias correction step is important to improve the accuracy of beta estimates. A few methods have been proposed for this purpose. One is the Illumina normalization method implemented in the Genome Studio software. For each sample, the Illumina method first divides all intensity values in each color channel by the average intensity value of the internal normalization control probes for that channel, and then rescales the intensity values using the first sample as the referent. Another method implemented in the Bioconductor package methylumi [12] is identical to the Illumina method, but instead of using the first sample, it uses as the referent the sample that has the smallest difference between the two color channel intensity values for the normalization control probes. The third method is All Sample Mean Normalization [14], or ASMN, which is also a modification of the Illumina method. It uses the “average” across all samples as the referent. The fourth and fifth methods are the nanes and nanet methods implemented in the Bioconductor package wateRmelon [10], which perform quantile normalization between methylated and unmethylated intensity values either separately for Infinium I and Infinium II probes (nanes) or jointly for all probes (nanet). The sixth and seventh methods are the smooth quantile normalization method and the simple scaling normalization method implemented in Bioconductor package lumi [5].

Here we propose REgression on Logarithm of Internal Control probes (RELIC), a novel dye-bias correction method based on data properties observed in paired internal normalization control probes. It is simple, accurate and efficient. We comprehensively evaluated the method in multiple datasets for robustness and accuracy and found that RELIC significantly outperformed the alternative methods. RELIC has been implemented in Bioconductor package ENmix [13], is freely available for use, and can be combined with other pre-processing packages that use minfi [1] data structure.

## Methods

### RELIC: REgression on Logarithm of Internal Control probes

The Illumina HumanMethylation450 BeadChip contains 93 pairs of internal normalization control probes (name with prefix NORM_A, NORM_T, NORM_G or NORM_C), while its successor MethylationEPIC BeadChip contains 85 pairs. The two probes in each pair are designed to target the same DNA region within housekeeping genes and contain no underlying CpG sites: one probe will extend to incorporate a base A or T (NORM_A or NORM_T, red channel), and the other probe will incorporate a base G or C (NORM_G or NORM_C, green channel). These probe pairs were designed to monitor array performance in different color channels and thus can be used for dye-bias correction. If there were no dye-bias, the intensity values from the two probes of each pair would be expected to be the same with a ratio close to 1.

Scatterplots of the log transformed intensity values between red and green channels for these normalization control probes demonstrates a clear linear relationship in every sample. A typical plot is shown in Additional file 1: Figure S1, which is for a normal breast tissue sample from [4] (GEO accession number: GSM815146). This motivates the new method, RELIC, which first performs a regression on the logarithms of the intensity values of the normalization control probes to derive a quantitative relationship between red and green channels, and then uses the relationship to correct for dye-bias on intensity values for whole array. Specifically, for each sample RELIC adjusts all intensity values from the green channel as:

$$ {I}_{i,adj}={e}^{{\widehat{\beta}}_1. \log \left({I}_i\right)+{\widehat{\beta}}_0}, $$

where *i* is the index of probe and *I*_*i*_ is the intensity value of the probe in the green channel and *I*_*i,adj*_ is the adjusted intensity value; ${\widehat{\beta}}_0$ and ${\widehat{\beta}}_1$ are linear regression coefficients from the regression analysis of the logarithms of intensity values between paired normalization control probes for the same sample (the logarithms of intensity values from the green channel, i.e., NORM_G and NORM_C, are modeled as independent variable). The intensity values from the red channel remain unchanged. One advantage of deriving the relationship between red and green channels using log transformed intensity values of normalization control probes is to assure non-negative values after the adjustment using the derived relationship.

### Evaluation datasets

Dataset 1: 450K dataset of a total of 39 methylation laboratory standard control samples reported by [13]. Human unmethylated DNA (HCT116 double knock out (DKO) of both DNA methyltransferases DNMT1 (-/-) and DNMT3b (-/-)) and fully methylated DNA (HCT116 DKO DNA enzymatically methylated) were obtained commercially (Zymo Research, Irving CA) and mixed together in different proportions to create laboratory control samples with specific methylation levels: 0, 5, 10, 20, 40, 50, 60, 80 and 100% methylated. Replicates for each methylation level (*n* = 10, 3, 2, 3, 3, 2, 3, 3 and 10, respectively) were independently assayed on different arrays.

Dataset 2: 450K dataset of 22 samples reported by [4]. These samples included three replicates from the HCT116 WT cell-line, three replicates from the HCT116 DNMT1 and DNMT3B double KO (DKO) cell-line, and 16 other samples (GEO accession number: GSE29290). In particular to evaluate RELIC and other dye-bias correction methods, we used the six replicates from the HCT116 WT and HCT116 DKO cell-lines, and the matched bisulfite pyrosequencing (BPS) data for 15 probes in the two cell-lines reported in the Table one of [4]. As described in [4] the fifteen CpGs were selected for technical validation of the 450K array measures (six sites for Infinium I assay and nine sites for Infinium II assay) using the more accurate BPS method as the “gold standard”.

Dataset 3: 450K dataset of 24 samples reported by [6]. These samples included 12 blood samples and 12 saliva samples for ten individuals, with two individuals having two technical blood/saliva replicates (GEO accession number: GSE73745). More specifically, we used these samples and the matched bisulfite pyrosequencing (BPS) data for three probes (cg19754622, cg16106427, cg08899523) to evaluate RELIC and other dye-bias correction methods.

## Results

### The dye-bias correction effect of RELIC

**Fig. 1:**
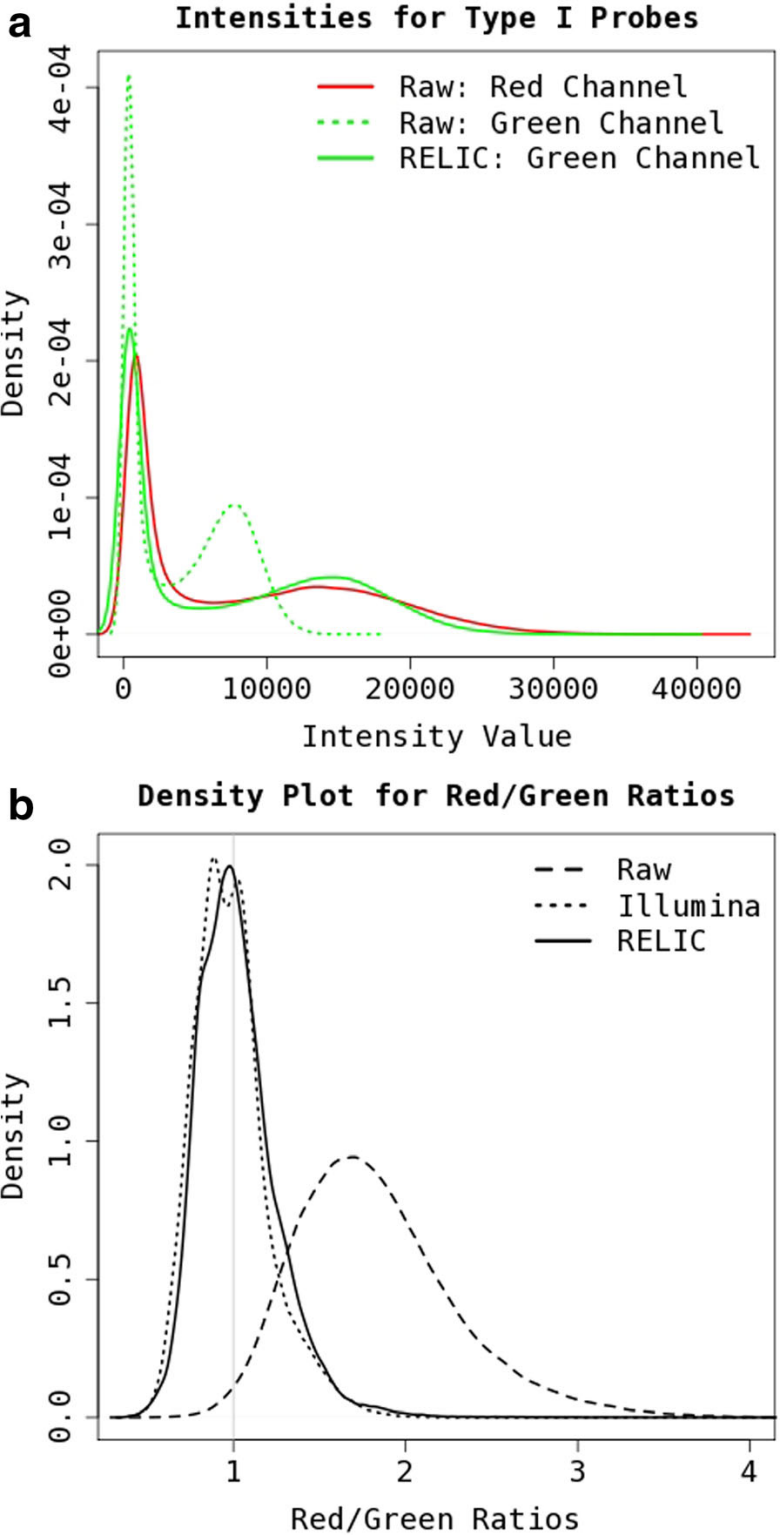
Demonstration of dye-bias correction effects with **a**) Intensity value distributions for Infinium I probes on red and green channels before and after adjustment with RELIC method for a typical sample; **b**) Distribution density plot for red/green ratios in normalization control probes for 889 blood samples before and after adjustment with RELIC or the Illumina method

To demonstrate RELIC dye-bias correction effect, we applied the method to a large 450K dataset with 889 blood samples [8]. Although dye-bias correction is more important for Infinium II probes, it is difficult to compare intensity distributions between red and green channels for Infinium II probes because each channel is for a specific methylation state and the overall quantitative distribution of methylated and unmethylated alleles would not be expected to be the same in every sample. In contrast, for Infinium I loci both methylated and unmethylated alleles are scanned in the same color channel (red for some loci, green for others). If there were no dye-bias, the distribution of combined methylated and unmethylated raw intensity values for Infinium I probes in the red channel (89,187 probes) should be similar to that of Infinium I probes in the green channel (46,289 probes). However, as shown in Fig. 1 they are very different, with compressed distribution in the green channel. After dye-bias correction with RELIC the distributions are more similar (Fig. 1a).

The different performance between Infinium I and II probes is partially due to dye-bias [3]. A correction for dye-bias thus can help reduce the beta value distribution difference between probe types (Additional file 1: Figure S2). We noticed that the red/green intensity ratios are not constant for different magnitude of intensity values. Scatter plot for the normalization control probes in all 889 samples showed a clear linear relationship in the log scale between red/green ratios and intensity values from the red channel (Additional file 1: Figure S3) with larger intensity values corresponding larger ratios. The Illumina method and its two variations (methylumi and ASMN) assume constant red/green ratios, which can result in under-adjustment at large intensity values and over-adjustment at small intensity values. This can be demonstrated in Fig. 1b -- the distribution density plot for red/green ratios after adjustment with the Illumina method has a bimodal peak which is overall less than 1. In contrast, the distribution after RELIC adjustment has a single mode close to 1. Larger intensity values will have a larger impact in calculating the arithmetic mean used in the Illumina method, which explains the slight left shifted (overall over-adjustment) distribution plot compared to the RELIC method (Fig. 1b). Furthermore, as shown in Additional file 1: Figure S4, a large percentage of actual intensity values are much smaller than the averaged intensity values for normalization control probes, and thus we would expect more severe overall over-adjustment with the Illumina method, especially among Infinium II probes with small beta values. This can be appreciated by the right shift on the left side of the beta value distribution for Infinium II probes in Additional file 1: Figure S2, resulting in further compression of the distribution among small beta values in Infinium II probes.

### Improvement on accuracy of beta estimates

To evaluate RELIC and compare it with alternative methods in terms of data quality improvement, we applied RELIC and six other dye-bias correction methods to the three datasets. The six alternative methods are: the Illumina method (Illumina), a modification of the Illumina method implemented in methylumi (methylumi), ASMN, the nanet method implemented in wateRmelon (nanet), the smooth quantile normalization implemented in lumi (lumi.quantile), the simple scaling normalization implemented in lumi (lumi.ssn). We also included results without adjustment (raw) for comparison. We did not include the nanes method (implemented in wateRmelon) in the comparisons as the nanet method is better than the nanes method, according to the Table two in [10].

#### Results for Dataset 1

**Fig. 2:**
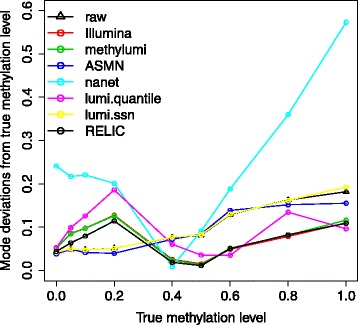
Effect of various dye-bias correction methods on methylation accuracy in Dataset 1, in which nine different methylation levels (0, 5, 10, 20, 40, 50, 60, 80 and 100%) were created by mixing unmethylated and fully methylated DNA together in different proportions. Shown are averaged absolute deviations of the beta value (adjusted using different methods) distribution mode from expected methylation level

Although all CpG sites in the experimental control samples are expected to have the same methylation level, the actual beta measurements often have skewed (non-normal) distribution (data not shown), and thus for each sample we used the beta value distribution mode as the methylation level estimate. For each of the seven dye-bias correction methods, we calculated the absolute difference between the expected methylation and the averaged methylation mode across replicates for each methylation level group (Fig. 2). The performances of ASMN and lumi.ssn are similar to raw data and the deviations from true values are linearly correlated with the magnitude of methylation level. Quantile normalization methods (nanet and lumi.quantile) have the highest variability depending on methylation level. In contrast, the performances for methods using normalization control probes (RELIC, Illumina and methylumi) are much more robust across different levels of methylation. Methods Illumina and methylumi have almost identical performance. RELIC performed slightly better than them, particularly when methylation levels are small, perhaps reflecting over-adjustment of small beta values by the other two methods. Overall, RELIC estimates were statistically significantly closer to the expected level than all other methods (Additional file 1: Table S1) with one-sided Student paired T test *p* values for the 39 samples range from 4.0 × 10^−3^ to 1.2 × 10^−11^. Separate evaluations demonstrated the same pattern (Additional file 1: Table S1) for Infinium I (*p* values between 4.5 × 10^−4^ and 7.3 × 10^−15^) and Infinium II probes (*p* values between 3.5 × 10^−3^ and 1.7 × 10^−11^).

#### Results for Dataset 2

**Fig. 3:**
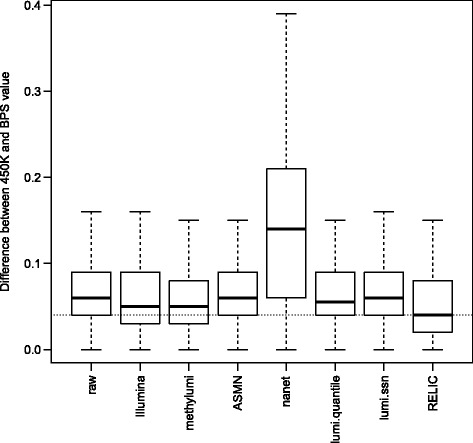
Effect of various dye-bias correction methods on methylation accuracy for Dataset 2 (GSE29290). Shown are boxplots of absolute differences between the beta values (adjusted using different methods) and the corresponding pyrosequencing values for 90 measurements

Bisulfite pyrosequencing (BPS) measurements were available to validate 450K array measures for 15 probes (six Infinium I probes and nine Infinium II probes) in Dataset 2. Similar to [4], we used the BPS validation data as the gold standard in our evaluation. For each of the six samples (three replicates from each of the two cell-lines (HCT116 WT and HCT116 DKO)), we calculated the absolute difference of beta values between the BPS measures and 450K array measures corrected by each dye-bias correction method at each of the fifteen probe sites. For the total of 90 measurements, RELIC outperforms other methods (Fig. 3) with statistically significantly smaller mean deviations from the gold standard measures (one-sided Student’s T test *p* values range from 7.7 × 10^−3^ to 3.2 × 10^−15^, as shown in Additional file 1: Table S1).

#### Results for Dataset 3

**Fig. 4:**
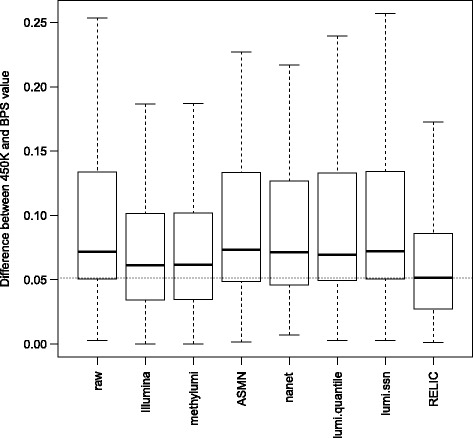
Effect of various dye-bias correction methods on methylation accuracy for Dataset 3 (GSE73745). Shown are boxplots of absolute differences between the beta values (adjusted using different methods) and the corresponding pyrosequencing values for 72 measurements

To determine whether the effects in Dataset 2 are sample specific, we further evaluated these methods in Dataset 3 that contains both 450K data and BPS data for 3 CpGs (one Infinium I probe and two Infinium II probes) in 24 samples and calculated the absolute difference of beta values between the gold standard BPS measures and the 450K estimates. For the total of 72 measurements, RELIC again outperformed other methods (Fig. 4) with one-sided Student’s T test *p* values ranging from 3.3 × 10^−4^ to 1.3 × 10^−16^ (Additional file 1: Table S1).

## Discussion

The two types of dye (Cy3-green/Cy5-red) employed in Illumina’s methylation arrays have different performance characteristics, which often result in relatively compressed data distribution in the green channel. The discrepancy between color channels can directly affect methylation beta value calculation, especially for Infinium II probes (accounting for 72% of probes in HumanMethylation450 array and 84% of probes in MethylationEPIC array) where methylated states are evaluated in green channel and unmethylated states are evaluated in red channel. Based on the observed data property that intensity values are linearly correlated at log scale between red and green channels, we here introduced a novel dye-bias correction method, RELIC, to reduce the discrepancy and improve data quality. We demonstrate that RELIC can efficiently extend intensity value distribution in the green channel so the adjusted values are comparable to red channel data.

We have evaluated all currently available dye-bias correction methods in several datasets (experimental controls, cell lines, blood and saliva samples), the results can be used to guide whole genome DNA methylation data analysis. To best demonstrate the effect of dye-bias correction methods, we intentionally avoided using additional preprocessing steps (background correction, inter-array normalization and probe type bias correction) that may normally be employed in Illumina DNA methylation array analysis. Our evaluations reveal that dye-bias correction methods based on internal normalization control probes perform better and more robust than quantile normalization methods that use all probe-sets on an array. This is because by design, in the absence of dye-bias, the two probes in each normalization control probe pair are expected to have identical measures. In contrast, it may not be valid to assume that intensity value distributions for red and green channels are identical in every biological sample. This is particularly true for Infinium II probes, where all methylated intensities are measured in the green channel and all unmethylated intensities are measured in the red channel. This can be demonstrated in Fig. 2, where the performance of the nanet method is poorest when expected beta values are either small (methylated intensity values (green) are much lower than unmethylated intensity values (red)) or large (methylated intensity values (green) are much higher than unmethylated intensity values (red)). The Illumina method and its variations are more robust. However, they assume constant red/green ratios, which can be over-adjusted for small red intensity values and can be under-adjusted for large red intensity values, resulting in further compressed beta value distribution on the left side, particularly for Infinium II probes.

We are unable to find EPIC data with bisulfite pyrosequencing validation, and thus we only evaluated these methods in 450K datasets. Although EPIC arrays have slightly fewer normalization control probe pairs, the application of the RELIC method is robust: using only a subset of normalization control probe pairs (85 random pairs from 93 pairs) we have done similar evaluations in Dataset 2 with results similar to that based on full normalization control probe pair set (data not shown). Thus we believe RELIC can reliably be applied in the analysis of EPIC data.

We also performed a comparison of different dye-bias correction methods in combination with three probe type bias correction methods: SWAN [7], BMIQ [11] and RCP [9], using Dataset 2. The results show that all probe type bias correction methods can further improve data accuracy (Additional file 1: Figure S5), and the improvement by RCP is greater than that by BMIQ, which is greater than the improvement by SWAN. Also, we saw that RELIC is one of the best methods among all dye-bias correction method no matter which probe type bias correction method is applied as the downstream step.

## Conclusions

We demonstrated that RELIC is a robust and efficient method to correct for dye-bias in Illumina Methylation BeadChip data, and that it statistically significantly outperforms all alternative methods, resulting in improved accuracy of methylation beta value estimates. We incorporated RELIC method into a user friendly R Bioconductor software package ENmix [13] to facilitate DNA methylation data analysis.

## Additional file

Additional file 1: Supplementary_Material. This docx file contains all supplementary tables and supplementary figures. (DOCX 424 kb)
