# Supplementary material for: RELIC: a novel dye-bias correction method for Illumina Methylation BeadChip
Source: BMC Genomics. 2017 Jan 3;18:4. doi: 10.1186/s12864-016-3426-3 (PMC5209853; doi:10.1186/s12864-016-3426-3)
Supplement: Additional file 1: — Supplementary_Material. This docx file contains all supplementary tables and supplementary figures. (DOCX 424 kb) [file 12864_2016_3426_MOESM1_ESM.docx]

**Title:** RELIC: a novel dye-bias correction method for Illumina Methylation BeadChip

Zongli Xu, Sabine A. S. Langie, Patrick De Boever, Jack A. Taylor and Liang Niu

**Additional file**

1. **Supplementary Tables**
2. **Supplementary Figures**
3. **Additional file**

**Table S1.** Comparison of RELIC to other dye-bias correction methods in terms of data accuracy improvement in three datasets: Dataset 1 (laboratory standard control samples), Dataset 2 (GSE29290) and Dataset 3 (GSE73745). Entries are p-values from one-sided Student paired T tests which test null hypotheses (RELIC and alternative methods have the same effect on data accuracy improvement) versus alternative hypotheses (RELIC leads to better data accuracy improvement). For Dataset1, the data used for the tests are the absolute differences between the beta (adjusted by RELIC or the other alternatives) distribution modes (obtained using all data, data from Infinium I probes, or data from Infinium II probes) and the expected methylation levels in 39 samples; for Dataset 2, the data used for the tests are the 90 absolute differences between beta values (adjusted by RELIC or the other alternatives) and the corresponding pyrosequencing values; for Dataset 3, the data used for the tests are the 72 absolute differences between beta values (adjusted by RELIC or the other alternatives) and the corresponding pyrosequencing values.

| Method | Dataset 1 (All) | Dataset 1 (Infinium I) | Dataset 1 (Infinium II) | | Dataset 2 | Dataset 3 |
| --- | --- | --- | --- | --- | --- | --- |
| raw | $3.3\times10^{-4}$ | $4.5\times10^{-4}$ | | $2.5\times10^{-4}$ | $1.1\times10^{-3}$ | $2.2\times10^{-13}$ |
| Illumina | $3.1\times10^{-4}$ | $2.5\times10^{-4}$ | | $3.5\times10^{-3}$ | $8.1\times10^{-5}$ | $3.3\times10^{-4}$ |
| methylumi | $2.2\times10^{-6}$ | $1.2\times10^{-4}$ | | $8.5\times10^{-5}$ | $4.0\times10^{-3}$ | $1.9\times10^{-4}$ |
| ASMN | $4.0\times10^{-3}$ | $2.7\times10^{-4}$ | | $3.4\times10^{-3}$ | $6.7\times10^{-4}$ | $1.9\times10^{-16}$ |
| nanet | $1.2\times10^{-11}$ | $7.3\times10^{-15}$ | | $1.7\times10^{-11}$ | $3.2\times10^{-15}$ | $7.5\times10^{-14}$ |
| lumi.quantile | $5.7\times10^{-4}$ | $2.4\times10^{-6}$ | | $6.4\times10^{-4}$ | $7.7\times10^{-3}$ | $1.3\times10^{-16}$ |
| lumi.ssn | $1.9\times10^{-4}$ | $1.2\times10^{-4}$ | | $1.5\times10^{-4}$ | $2.2\times10^{-4}$ | $2.6\times10^{-13}$ |

1. **Additional file**

**Figure S1.** Scatterplot of the log transformed intensity values between red (AT controls) and green channels (GC controls) for normalization control probe pairs in the GEO sample GSM815146. Dashed line is $y=x$, all points would be expected to be on this line if there was no dye-bias.

**Figure S2.** Beta value distribution for Infinium I and Infinium II probes before and after adjustment with RELIC or the Illumina method for a typical blood sample from (Markunas, et al., 2014).
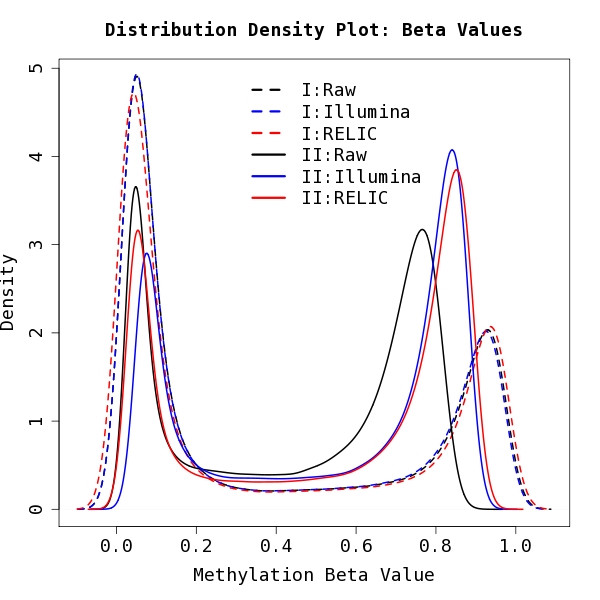


**Figure S3**. Scatter plot for red/green ratios versus intensity values from red channel (in log scale). The Plotted are for all internal normalization control probes in Illumina 450K methylation data of 889 blood samples from (Markunas, et al., 2014). Red/green ratio is calculated using intensity values from red and green channels for each paired control probe. Color indicates density of dots with red for most densely distributed dots and light blue for least densely distributed dots. If absence of dye-bias, the log ratios of log(red/green) is expected to be zero. Most ratios from the real dataset are deviated from zero, and are linearly corrected with red channel intensity at log scale.


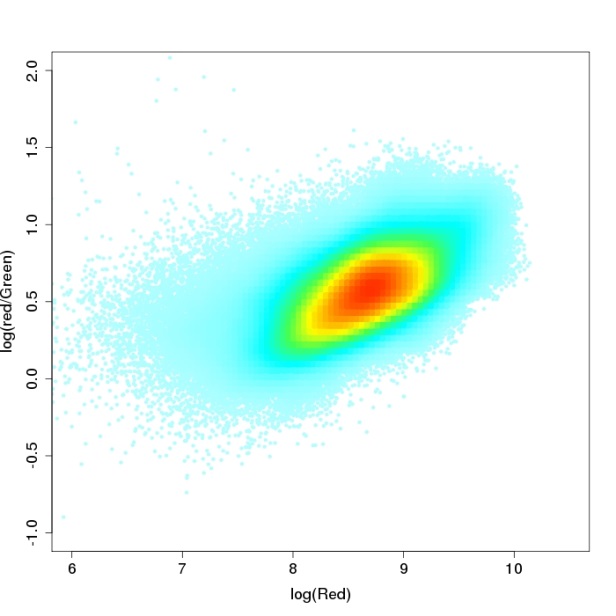


**Figure S4.** Intensity distribution plots at red or green channel for normalization control probes (black) and array probes (red or green).


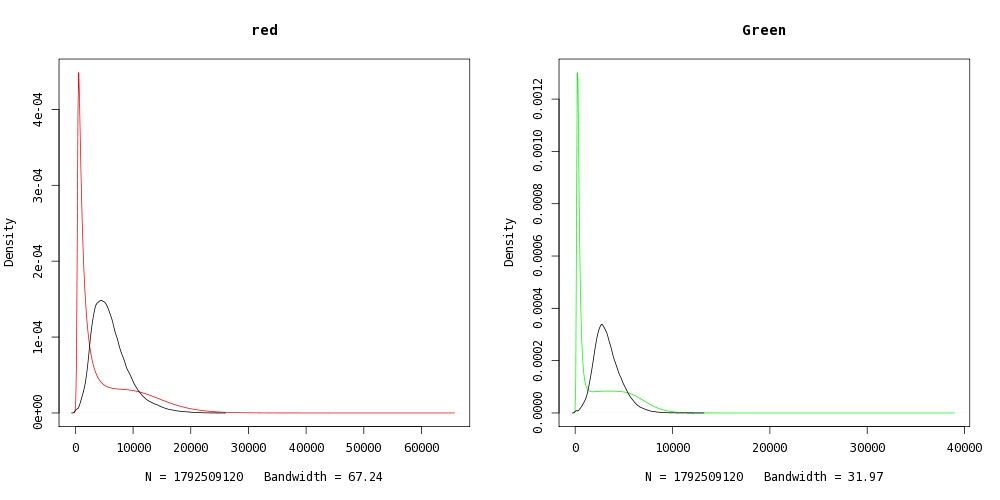


**Figure S5.** Comparison of dye-bias correction methods when they are combined with three probe type bias correction methods (SWAN, BMIQ and RCP) using Dataset 2. For each combination of dye-bias correction method and probe type bias correction method, the mean of the 90 absolute differences between the adjusted beta values and the BPS values was shown as a bar in the figure.

Markunas, C.A.*, et al.* Identification of DNA methylation changes in newborns related to maternal smoking during pregnancy. *Environ Health Perspect* 2014;122(10):1147-1153.
